# Medium to Long-Term Clinical Outcomes of Spinal Metastasectomy

**DOI:** 10.3390/cancers14122852

**Published:** 2022-06-09

**Authors:** Satoshi Kato, Satoru Demura, Hideki Murakami, Kazuya Shinmura, Noriaki Yokogawa, Ryohei Annen, Motoya Kobayashi, Yohei Yamada, Satoshi Nagatani, Norio Kawahara, Hiroyuki Tsuchiya

**Affiliations:** 1 Department of Orthopaedic Surgery, Graduate School of Medical Sciences, Kanazawa University, Kanazawa 920-8641, Japan; msdemura@gmail.com (S.D.); kazuyashinmura@yahoo.co.jp (K.S.); chakkun1981chakkun@yahoo.co.jp (N.Y.); r_annen@mac.com (R.A.); kobamoto1220@gmail.com (M.K.); y.ymd.0aw@gmail.com (Y.Y.); snag0806@gmail.com (S.N.); tsuchi@med.kanazawa-u.ac.jp (H.T.); 2 Department of Orthopaedic Surgery, Graduate School of Medical Sciences, Nagoya City University, Nagoya 467-8601, Japan; hmuraka@med.nagoya-cu.ac.jp; 3 Department of Orthopaedic Surgery, Kanazawa Medical University, Kahoku 920-0293, Japan; kawa@kanazawa-med.ac.jp

**Keywords:** clinical outcome, complication, metastasectomy, spinal metastasis, survival rate

## Abstract

**Simple Summary:**

With the prolonged survival of metastatic cancer patients, medium to long-term outcomes of local therapy for oligometastases are important and should be discussed. Metastasectomy is a reasonable treatment for isolated lesions when feasible. This retrospective study aimed to examine the medium to long-term outcomes of spinal metastasectomy in 124 patients with isolated spinal metastases who underwent metastasectomy. The primary malignancy was renal cell carcinoma in 51 patients, thyroid carcinoma in 14 patients, low-grade sarcoma in 13 patients, lung cancer in 13 patients, breast cancer in 12 patients, and other malignancies in 21 patients. The 3-year and 5-year survival rates were 70% and 60%, respectively. Patients with thyroid cancer had the best survival results. The occurrence rates for the local recurrence of the operated spinal lesion and instrumentation failure were 10% and 22%, respectively. For certain patients with isolated and removable spine metastases, metastasectomy can be a useful treatment option.

**Abstract:**

The prolonged survival of metastatic cancer patients highlights the importance of the local control of spinal metastases, which reduce patient performance status. This retrospective study examined the medium to long-term outcomes of spinal metastasectomy by evaluating 124 patients who underwent metastasectomy for isolated spinal metastases (2006–2018) with a postoperative follow-up for a minimum of 3 years. The findings present information on patient demographics (i.e., performance status, location of non-spinal metastases, and history of systemic therapy) and postoperative outcomes, including perioperative complications, disease progression of non-operated metastases, and additional excisional surgeries. Additionally, postoperative survival, local tumor control in the operated spine, and maintenance of spinal reconstruction without instrumentation failure were determined using Kaplan–Meier analyses. The primary malignancy was kidney and thyroid cancer in 51 and 14 patients, respectively, low-grade sarcoma and lung cancer in 13 patients, breast cancer in 12 patients, and other malignancies in 21 patients. The 3-year and 5-year survival rates were 70% and 60%, respectively. We found that patients with thyroid cancer had the best survival results, with local tumor recurrence and instrumentation failure at 10% and 22%, respectively. These findings suggest that for certain patients with isolated and removable spine metastases, metastasectomy can improve function and survival.

## 1. Introduction

The skeleton is the third most common site of metastases after the lungs and liver, with the spine being the most frequently affected bone [1]. Spinal metastases (SMs) occur in 20–40% of patients with cancer, with up to 20% of them becoming symptomatic [2,3,4]. SMs often result in severe pain and neurological symptoms, which reduce performance status (PS) and increase patient mortality, resulting in severe pain and neurological symptoms, even with the use of conservative therapies [5,6]. In general, skeletal metastases from solid tumors are more resistant than visceral metastases to systemic therapies, including chemotherapy, molecular-targeted therapy, and immunotherapy [7]. In patients with SMs, a decrease in PS not only directly affects mortality but also affects it indirectly by preventing the application of systemic therapies. Based on these factors, the local control of SMs is very important for patients with SMs, and surgical resection is a reasonable treatment option for isolated lesions when feasible [8]. However, the use of metastasectomy for SMs has been limited owing to the high technical demands and significant complications associated with the procedure [9]. Therefore, the surgical treatment of SMs is generally palliative in order to maintain or improve patient PS and quality of life (QOL). It is intended to provide pain relief and preserve or restore neurologic function, although it is often effective only in the short term [10].

**Figure 1:**
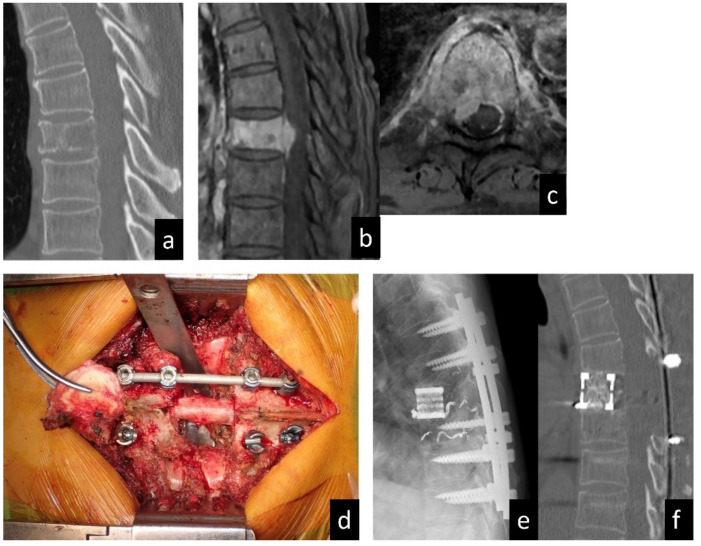
A 63-year-old man diagnosed with SM of T8 from renal cell carcinoma. (**a**) Sagittal plane computed tomography image showing the osteolytic lesion in the T8 vertebral body. (**b**) Sagittal and (**c**) axial images of enhanced T1-weighed magnetic resonance imaging showing spinal canal extension of the tumor. (**d**) Intraoperative photo in the resected vertebral body. (**e**) Radiograph in lateral view and (**f**) computed tomography in sagittal view showing the reconstructed spine after metastasectomy (total en bloc spondylectomy).

Spinal metastasectomy (complete excision of spinal lesions) is challenging and technically demanding because the spine contains the spinal cord and nerve roots and is in close proximity to major vessels and vital organs. In conventional excisional surgery for SMs, piecemeal excision is commonly practiced. One of the biggest disadvantages of this procedure is the high risk of residual tumor tissue in the vertebra and the surrounding structures at the site, which can lead to a higher rate of local tumor recurrence. Spondylectomy (vertebrectomy), a complete resection of the tumor-affected vertebrae, was first reported by Stener [11]. In the 1990s, Tomita et al. reported on total en bloc spondylectomy (TES), which aimed to achieve a complete resection of spinal tumors while reducing the risk of tumor recurrence and complications (Figure 1) [12]. However, spondylectomy is still an extraordinary type of surgery among general spine surgeons, and several authors have reported a higher rate of perioperative complications [13,14,15,16]. Improvements in TES surgical techniques over the years have enabled surgeons to achieve good outcomes with lowered significant complications [17,18,19]. In most patients with SMs who are candidates for spinal metastasectomy, the tumor involves at least one pedicle with an epidural spinal cord compression. During the spinal metastasectomy of such cases, an intralesional transpedicular osteotomy using a fine thread wire saw should be carried out to prevent spinal-cord and nerve-root injuries [12,17,18]. Although the procedure is not marginal (R0 resection) in this situation, it is highly practical, and most spine surgeons employ this technique to achieve the complete resection of the tumor-affected vertebra [8]. Recently, the number of publications on spinal metastasectomy has been increasing [14,15,16,20,21,22,23,24,25,26,27], mostly with the reporting of short-term outcomes. With the prolonged survival of patients with metastatic tumors due to effective and modern treatments, medium to long-term clinical outcomes of local therapy, including late complications, and disease conditions in both treated and untreated sites are important and should be discussed. We hypothesized that spinal metastasectomy in a high-volume center affords long-term PS maintenance and future opportunities for effective therapies, and that it can improve survival. This study examined the medium to long-term clinical outcome of spinal metastasectomy, including postoperative survival rate, late complications, local recurrence in the operated site, and disease conditions in the non-operated site. Based on the outcomes, this article will also discuss the indications of spinal metastasectomy according to cancer types.

## 2. Patients and Methods

### 2.1. Patient Selection

After obtaining approval from the Institutional Review Board of our University (IRB number: 2015-075), a database of SM patients who underwent spinal metastasectomy at our university hospital between 2006 and 2018 was retrospectively reviewed at the end of 2021. Follow-up data for a minimum of 3 years were obtained for SM patients. Since 2006, all imaging tests and their reports from radiologists have been stored in the electronic medical records of our institute. Up to 3 years after surgery, patients were followed up at least every 6 months during outpatient visits or by telephone survey for patients who lived too far away to visit the clinic. From 3 years after surgery onwards, follow-up was continued every 6 or 12 months, until the patient died. During outpatient visits, computed tomographies (CTs) of the area from the chest to the pelvis were routinely performed to examine previously recognized metastases and to detect new metastases. At our institution, spinal metastasectomy was indicated based on the following criteria: solitary and removable lesion in the spine (tumors involving ≤ 3 contiguous vertebrae), operability (Eastern Cooperative Oncology Group (ECOG) PS ≤ 3), and stable disease with no or a limited number of other metastases. Informed consent for surgery was obtained from the patients. In this study, patients who had isolated spinal lesions and underwent metastasectomy for the lesions were enrolled regardless of existing extraspinal metastases at the time of surgery. Patients who were lost to postoperative follow-up within 3 years after surgery were excluded from this study.

### 2.2. Features Studied

Survival was defined as the time from spinal metastasectomy until death or the last follow-up, and it was compared among the malignancy types. Local tumor recurrence and instrumentation failure in the operated spine after surgery were evaluated using multiplanar reconstruction computed tomography (CT). When a local tumor recurrence in the operated lesion was suspected by CT, every patient had to undergo magnetic resonance imaging to confirm the presence of recurrent tumors. The other metastases which were preoperatively identified or developed postoperatively were also evaluated using chest-to-pelvis CT. The treatments for other bone metastases were examined and compared according to malignancy types. The revised Tokuhashi [28] and Tomita [29] scores at the time of the metastasectomy for the SMs were also examined as representative prognostic scoring systems.

For the patients who survived 3 years or more after the metastasectomy of the spine lesions, we examined their PS, their history of systemic therapy and antiresorptive therapy with bisphosphonate or denosumab, and the disease progressions of bone metastases, except for the operated spine lesions and non-skeletal metastases for 3 years after surgery. In our institute, when the patients did not have any other bone metastases after spinal metastasectomy, antiresorptive therapy was not administered after surgery. Disease progression was defined as the presence of a new lesion, the enlargement of a previously identified lesion, or an indication of excisional surgery for the lesions. For the patients with disease progression, we examined whether they experienced additional excisional surgery for the lesions.

### 2.3. Statistical Methods

Continuous variables are expressed as mean ± standard deviation, and survivals are expressed as median. The Shapiro–Wilk test was used to assess the normality of data distribution. In the comparisons of the variables among the five malignancy groups, the Tukey or Games–Howell tests were used. Categorical data were expressed as frequencies and percentages, and comparisons between groups were made using the chi-squared test. The Kaplan–Meier analysis was used to calculate the postoperative survival for all patients, and the log-rank test was used to compare the survival rates among patient groups in univariate analyses. The Kaplan–Meier analysis was also used to calculate local tumor control in the operated spine and the maintenance of spinal reconstruction without instrumentation failure. All statistical analyses were performed using SPSS version 25 (IBM Corp., Armonk, NY, USA). *p*-values < 0.05 were considered statistically significant.

## 3. Results

**Figure 2:**
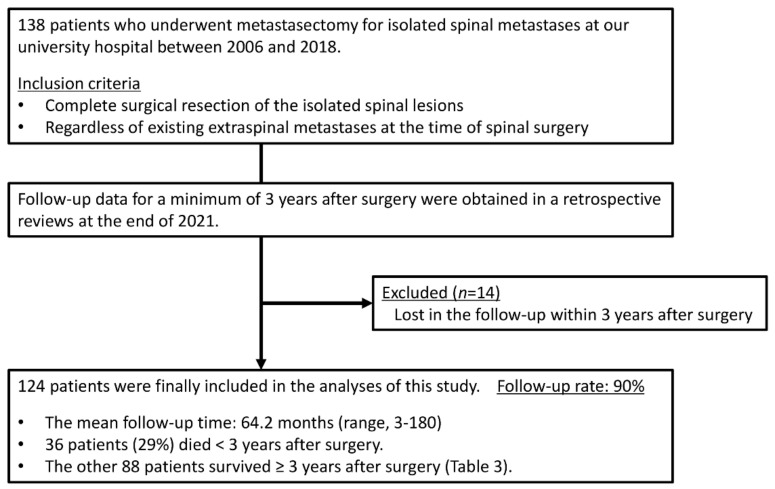
Study flow chart.

Fourteen of the 138 patients who underwent metastasectomy for isolated SMs were lost to follow-up within 3 years after surgery and therefore excluded from this study (follow-up rate: 90%). The study cohort included 124 patients (73 men and 51 women) with a mean age of 56.7 years (Figure 2). They were all Asian. The primary malignancy was renal cancer in 51 patients, thyroid cancer in 14 patients, low-grade sarcoma in 13 patients, lung cancer in 13 patients, breast cancer in 12 patients, and other malignancies in 21 patients (Table 1). Low-grade sarcomas included eight leiomyosarcomas, three chondrosarcomas, and two liposarcomas. Other malignancies included three colorectal cancers, three cancers of unknown primary origin, two bladder cancers, two esophageal cancers, two gastrointestinal stromal tumors, and nine others. Primary tumors were resected before or after spinal metastasectomy, except in three lung cancer and three other cancer (primary unknown) patients. The female ratio among the breast cancer patients was 100%, and that among the thyroid cancer patients was the second largest from among the five malignancy groups. All patients had isolated SMs in the thoracic or lumbar spine, and two-thirds had thoracic SMs. Sixty-eight patients (55%) had extraspinal metastases at the time of TES for SMs. Twenty-nine patients (23%) had lung lesions, and thirty-two patients (26%) had non-spinal bone lesions. Non-spinal bone lesions were mainly found in the pelvis in 16 patients, in the rib in 8 patients, and in the scapula in 6 patients. The patients with SMs from low-grade sarcoma or lung cancer had a lower revised Tokuhashi score and a higher Tomita score as compared with other patients owing to the scoring points of primary malignancy in both scoring systems. The scores implied that the postoperative survival was expected to be shorter. More than 50% of the patients underwent pre- and postoperative systemic therapies. Thirty-two patients (26%) had a history of radiotherapy for SMs before spinal metastasectomy. In our institute, postoperative radiotherapy was not employed after spinal metastasectomy. For patients with local tumor recurrence in the operated spine after spinal metastasectomy, radiotherapy with or without additional surgery was considered.

To achieve a complete oncological resection of the spinal lesions, vertebrectomy (complete resection of the whole diseased vertebra: TES) was performed in 116 patients and hemivertebrectomy (complete resection of the metastatic lesion with the preservation of the healthy part of the vertebral body) in 8 patients. Eighty-four patients underwent a single vertebral resection, 22 underwent two consecutive vertebral resections, and 18 had three consecutive vertebral resections. A single posterior approach was employed in 95 patients, and an anterior–posterior combined approach was employed in 29 patients. Two of the 29 anterior–posterior combined surgeries were two-stage surgeries. Twenty-two patients experienced major perioperative complications, including surgical site infection requiring surgical treatment (8 patients), significant neurological deterioration in extremities after surgery (5 patients), pleural effusion or pneumothorax requiring chest tube insertion (4 patients), pulmonary embolism (3 patients), intraoperative major vessel injury (1 patient), and spinal cord infarction associated with preoperative arterial embolization (1 patient). No patients had operation-related deaths.

**Figure 3:**
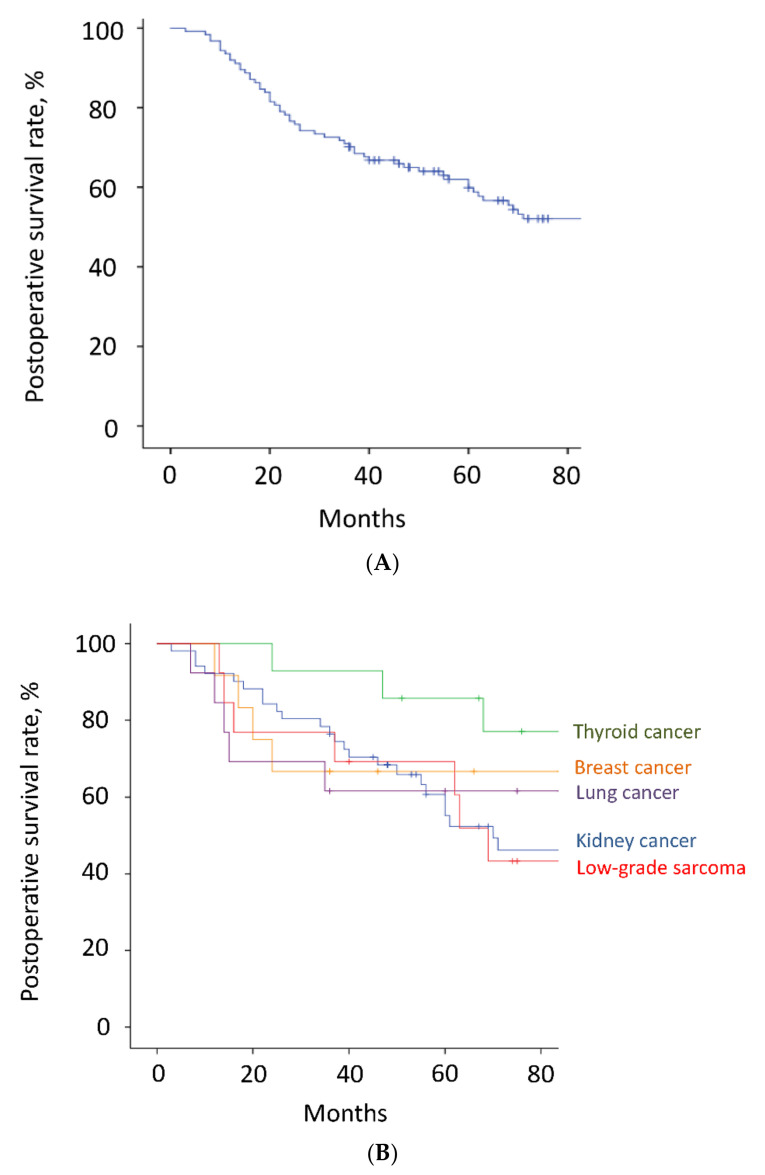
(**A**). Postoperative survival. Of all 124 patients, 36 patients died <3 years after surgery, whereas the other 88 patients survived ≥3 years after surgery. The 3-year and 5-year survival rates were 70% and 60%, respectively. The estimated median survival time was 93 months. The tick marks indicate the last date of follow-up. (**B**). Postoperative survival. The 3-year and 5-year survival rates of the patients with kidney cancer, thyroid cancer, low-grade sarcoma, lung cancer, and breast cancer were 77% and 61%, 93% and 86%, 77% and 69%, 62% and 62%, and 67% and 67%, respectively. The tick marks indicate the last date of follow-up.

**Figure 4:**
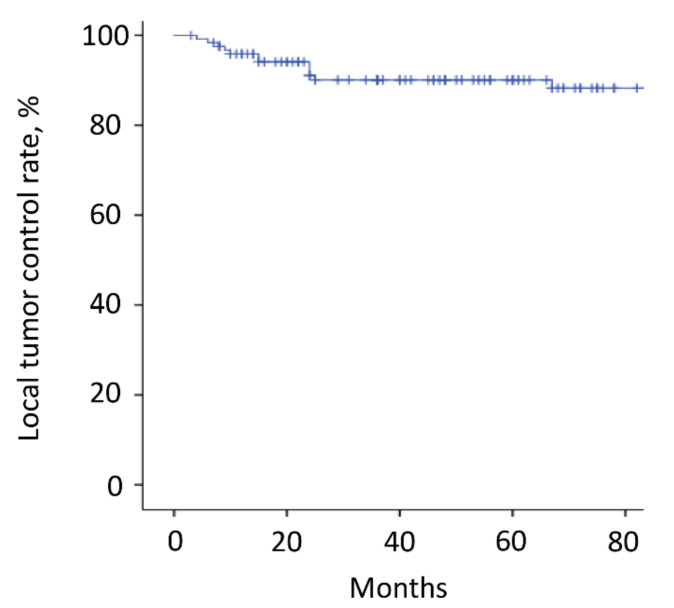
Local tumor recurrence. The local recurrence rate of the operated spinal lesion was 10% (12 patients). The 3-year and 5-year local tumor control rates were both 90%. All but one patient (11 patients) had a local tumor recurrence in the operated spine within 3 years after surgery. The tick marks indicate the last date of follow-up.

**Figure 5:**
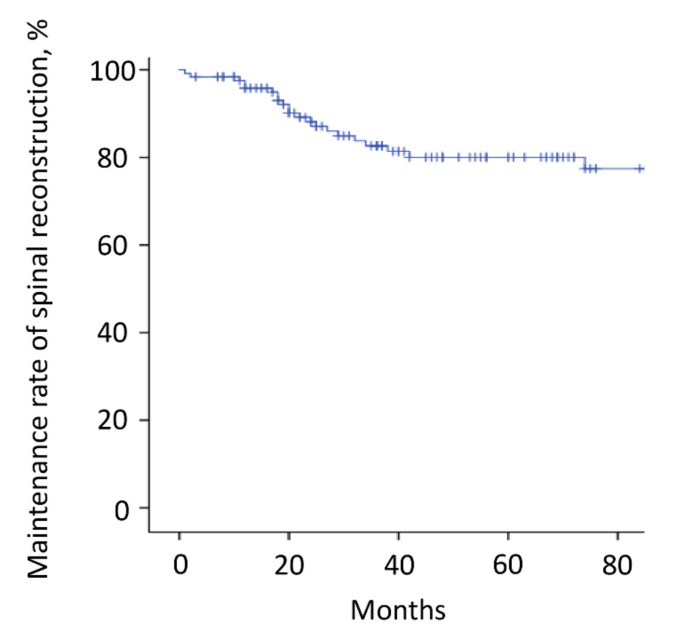
Instrumentation failure. The instrumentation failure rate requiring revision surgery was 22% (27 patients). The 3-year and 5-year maintenance rates of spinal reconstruction without instrumentation failure were 83% and 80%, respectively. Nineteen patients (70%) had the revision surgery within 3 years after metastasectomy. The tick marks indicate the last date of follow-up.

The mean follow-up time was 64.2 months (range, 3–180). Thirty-six patients (29%) died <3 years after surgery, whereas the other 88 patients survived ≥3 years after surgery. Overall, the 3-year and 5-year survival rates were 70% and 60%, respectively (Figure 3A and Table 2). The estimated median survival time was 93 months. The 3-year and 5-year survival rates of patients with kidney cancer, thyroid cancer, low-grade sarcoma, lung cancer, breast cancer, and other malignancies were 77% and 61%, 93% and 86%, 77% and 69%, 62% and 62%, 67% and 67%, and 43% and 43%, respectively (Figure 3B and Table 2). Although there were no significant differences between the groups, patients with thyroid cancer had the best survival results. Overall, the local recurrence rate of the operated spinal lesion was 10% (12 patients). Although there were no significant differences in the rates between the groups, the highest rate was observed in patients with low-grade sarcoma. The mean duration between surgery and local recurrence was 19.3 ± 16.2 months. Overall, the local tumor control rates were 90% after both 3 and 5 years (Figure 4). All 11 patients, except one, had a local tumor recurrence in the operated spine within 3 years after surgery. Three patients underwent additional excisional surgery, and seven patients underwent palliative surgery for spinal cord compression by recurrent tumors. Overall, the instrumentation failure rate requiring revision surgery was 22% (27 patients). Fifteen and twelve patients underwent single and multilevel vertebral resections, respectively. Twenty and seven patients underwent spinal metastasectomy in the thoracic and lumbar spine, respectively. Instrumentation failure was not correlated with the spinal level of metastasectomy nor the number of resected vertebrae. The mean duration between surgery and revision surgery for instrumentation failure was 40.1 ± 38.7 months. Overall, the 3-year and 5-year maintenance rates of spinal reconstruction without instrumentation failure were 83% and 80%, respectively (Figure 5). Nineteen patients (70%) had the revision surgery within 3 years after metastasectomy. None of the patients underwent reoperation for re-instrumentation failure.

For the 56 patients who had only one metastasis in the operated spine at the time of surgery, the 3-year and 5-year survival rates were 63% and 57%, respectively. For the other 68 patients who had non-spinal metastases, the 3-year and 5-year survival rates were 77% and 66%, respectively. The results were not different between the patients with or without other metastatic lesions. Of the 56 patients with only one metastasis, 24 (43%) survived with no evidence of disease for 3 years after spinal metastasectomy. The proportion of patients with no evidence of disease 3 years after surgery was the highest in patients with thyroid cancer (80%), followed by patients with breast (60%) and kidney (56%) cancer.

Table 3 shows the 3-year clinical outcomes of patients that survived for more than 3 years after metastasectomy for spine lesions. Eighty-eight patients (71%) survived ≥3 years after surgery. In >90% of the patients, PS remained good, with a grade of 0 or 1 by ECOG PS 3 years after surgery. Thirty patients (34%), especially those with breast cancer, received antiresorptive therapy as a postoperative course for 3 years. Twenty-seven patients (31%) had progression of bone metastases, except for the operated spine lesion within 3 years after surgery. More than one-third of the patients with kidney and breast cancers had this type of disease progression. Although most of the patients with kidney and thyroid cancers underwent additional excisional surgery for other progressive bone metastases, no patients with lung and breast cancers and low-grade sarcomas had the surgery. Thirty-six patients (40.9%) had a progression of non-skeletal metastases within 3 years after surgery. These patients were more likely to suffer from low-grade sarcoma and least likely to suffer from thyroid cancer. Seven (37%) and three (38%) patients with kidney cancer and low-grade sarcoma, respectively, underwent additional excision surgery for the non-skeletal metastases.

## 4. Discussion

Generally, the main treatment goals for patients with SMs are symptom palliation and maintenance or the improvement of PS and QOL. Conventional radiotherapy has been used as the primary and adjuvant treatment for SMs for decades. Palliative surgery is mainly indicated in the presence of pathological or impending fracture risk and spinal cord compression with or without vertebral fracture. In palliative surgery, the reconstruction or fixation of the diseased lesion is the main procedure, and spinal cord decompression with a partial resection of the tumor and/or laminectomy is also applied. Antiresorptive therapy is the current standard of care for preventing skeletal-related events in patients with bone metastases. In this study, 34% of patients who survived for more than 3 years after spinal metastasectomy, especially those with breast cancer, received antiresorptive therapy postoperatively. For oligometastatic disease, spinal stereotactic body radiotherapy (SBRT) has recently become the mainstay treatment for the long-term control of spinal metastases [7]. For patients with epidural diseases, separation surgery focused on spinal cord decompression is carried out to create a target for radiation prior to SBRT [30].

Despite the increasing efficacy of other modern systemic therapies, metastasectomy has become more frequently performed for some cancer types and conditions in the last few decades [31,32,33,34,35,36,37,38,39]. In the recent decades, metastasectomy has been reported most frequently for lung lesions that have easier surgical accessibility with fewer major complications [36,37,38], and the potential survival benefits of pulmonary metastasectomy have been increasingly recognized in the literature [39]. In contrast, spinal metastasectomy is particularly difficult and technically challenging owing to its anatomical characteristics, such as the encircling spinal cord and nerve roots and their proximity to large blood vessels. Therefore, medium or long-term clinical outcomes of studies on spinal metastasectomy with a significant sample size of >100 cases have not been reported. In the present study, with a total of 124 patients who underwent spinal metastasectomy, the postoperative survival rates were favorable, with 3-year and 5-year survival rates of 70% and 60%, respectively. Although there were no statistical differences between the malignancy types, the best survival result was observed in patients with thyroid cancer, followed by patients with low-grade sarcomas and kidney cancer 3 years postoperatively. Skeletal metastases from kidney and thyroid cancers and low-grade sarcomas have the following common features: (1) they are more resistant to systemic therapy and radiation than skeletal metastases from other major types of malignancy [6,40,41], (2) they correspond to the most destructive osteolytic lesions [6,40,41], (3) surgical resection of isolated metastatic lesions, when feasible, has been approved in prior studies or guidelines [42,43,44]. Therefore, isolated SMs that originate from these malignancies have a good indication for metastasectomy, and favorable outcomes have been reported even in patients with coexisting lung metastases [23,45].

Previously, curative-intent local therapy for metastatic lesions was not considered for patients with advanced non-small cell lung cancer (NSCLC) because of their poor prognosis. However, patients with oligometastases have a better prognosis than patients with more advanced NSCLC, and thus they may benefit from systemic therapy combined with curative-intent local therapy for metastatic lesions [46]. Several current guidelines recommend curative local therapy for patients with selected oligometastases [47,48].

Breast cancers are common malignancies that develop skeletal metastases, which tend to develop multiple lesions and are usually effectively treated with systemic and radiation therapy. However, in this study, three of the five patients with breast cancer who had only one metastasis in the spine at the time of surgery survived for 3 years or more with no evidence of disease. The strategy of the complete resection of metastases followed by observation has the advantages of not only avoiding the side effects of systemic drugs in patients, but also preserving effective drugs that can be used if the disease progresses in the future [34]. Based on the results of this study, although the surgical indication of metastasectomy for lung and breast cancer patients should be more stringent than that for kidney and thyroid cancer and low-grade sarcoma patients, spinal metastasectomy can be one of the treatment options for when patients have only one metastasis in the spine that is symptomatic, and surgical intervention can be considered for the lesion.

In this study, there was no difference in postoperative survival between patients who had only one metastasis in the spinal lesion they operated on and those who had a small number of metastases outside the spine. Recent studies have evaluated the role of metastasectomy combined with checkpoint and targeted therapies for oligometastases; these studies reported improved survival and sustained disease control in patients who underwent metastasectomy of oligometastatic lesions after the therapies [32,34,49]. Current trends suggest that prolonged survival with effective systemic therapy may allow additional opportunities for effective surgical treatment [32].

In this study, which had a medium or long-term follow-up, the local recurrence rate of the operated spinal lesion was 10%. In the case of solitary metastasis or oligometastases in the spine, spinal SBRT has been the principal treatment for the long-term control of SMs in recent years. Studies with large cohorts have reported one-year local control rates of 72–90% for various types of primary cancers, including radiosensitive tumors [50,51,52,53,54]. However, for spinal metastasectomy at well-experienced and high-volume centers, a lower local recurrence rate was reported as compared to that of spinal stereotactic body radiotherapy [8]. Additional surgeries for instrumentation failure were not rare, presenting an incidence rate of 22%. Previous studies have reported risk factors of instrumentation failure after TES, including case subsidence, the smaller number of instrumented vertebrae, high body mass index, history of radiotherapy, and lumbar level [55,56,57]. However, the complication did not affect the patient’s PS or prognosis because the clinical outcomes of the revision surgery for spinal reconstruction were favorable without a second instrumentation failure [58].

Among the patients in this study who survived for more than 3 years after spinal metastasectomy, >90% maintained their PS 3 years after surgery. Long-term local control by spinal metastasectomy afforded PS maintenance in patients with isolated spinal lesions. This condition provided the patients with the opportunity to receive future effective treatments. In this study, approximately 30% of the patients had disease progression of other bone metastases within 3 years post-operation. While most of the patients with kidney and thyroid cancers underwent additional metastasectomy, no patients with breast cancer had another surgery. This result can be explained by the fact that kidney and thyroid cancers are purely osteolytic, and guidelines have recommended metastasectomy for the removable skeletal lesions derived from these malignances. In contrast, skeletal metastases from breast cancer can be controlled by non-operative treatments, including antiresorptive therapy when they are detected in a non-symptomatic state during checkup.

Patient selection is essential for an appropriate surgical indication and favorable results. Based on this study and the literature [8,31,42,43,44], patients with thyroid and kidney cancer, low-grade sarcoma, and with isolated and removable spinal metastases are good candidates for spinal metastasectomy. In the case of lung and breast cancers, the indications for spinal metastasectomy are more stringent and should be limited to patients with significant symptoms from isolated and osteolytic spinal metastases that are refractory to conservative therapy and for whom surgical treatment for skeletal-related events is considered. Moreover, spinal metastasectomy should only be performed in experienced and high-volume centers because surgical feasibility with a lowered risk of perioperative complications is important for driving the decision of surgery.

The limitations of the present study include the retrospective collection of data of a heterogenous cohort from a single institute, which is a high-volume center for spine tumor surgery. Therefore, the results of this study cannot be directly applied to most hospitals and patients. Surgical feasibility with a low risk of perioperative complications is an important factor that drives the decision of surgery. Spinal metastasectomy should be performed only in well-experienced and high-volume centers. On the other hand, the number of spine surgeons who can perform the surgery has been increasing all over the world [14,15,16,20,21,22,23,24,25,26]. This study includes analyses of only the patients undergoing spinal metastasectomy without any controls, which could introduce biases. In contrast, this is the largest study to examine the medium or long-term clinical outcomes of spinal metastasectomy. We could not obtain detailed information about the indication of pre- and postoperative systemic therapies because the therapies by primary physicians were mostly performed at other hospitals (patients were referred to our institute for the purpose of spinal metastasectomy). Despite these limitations, this study provided informative clinical outcomes of spinal metastasectomy and indicated that patients with isolated and removable spinal metastases can benefit from metastasectomy, with the appropriate selection of patients.

## 5. Conclusions

In this study of 124 patients with isolated spinal metastases, the 3-year and 5-year survival rates after spinal metastasectomy were 70% and 60%, respectively. Patients with thyroid and kidney cancers and low-grade sarcomas had the best 3-year postoperative survival rates. Local tumor recurrence was low, which was at 10% in 5 years. For certain patients with isolated and removable spine metastases, metastasectomy is a useful treatment option as it can improve function and survival.

## Figures and Tables

**Table 1:** Background characteristics of the patients who underwent spinal metastasectomy.

Groups	Total	Kidney	Thyroid	Low-Grade Sarcoma	Lung	Breast	*p*
Number of patients	124	51	14	13	13	12	-
Age (year), mean (SD)	56.7 (10.7)	59.2 (9.1)	57.4 (9.5)	51.5 (11.4)	57.2 (8.2)	56.8 (10.2)	-
Sex, female, *n* (%)	51 (41.1)	9 (17.6)	12 (85.7)	5 (38.5)	7 (53.8)	12 (100)	<0.05
Level of metastatic lesion, *n* (%)							0.48
Thoracic	82 (66.1)	28 (54.9)	11 (78.6)	9 (69.2)	9 (69.2)	8 (66.7)	
Lumbar	42 (33.9)	23 (45.1)	3 (21.4)	4 (30.8)	4 (30.8)	4 (33.3)	
Preoperative ECOG PS, *n* (%)							0.93
0–1	89 (71.8)	35 (68.6)	11 (78.6)	9 (69.2)	10 (76.9)	9 (75.0)	
2–3	35 (28.2)	16 (31.4)	3 (21.4)	4 (30.8)	3 (23.1)	3 (25.0)	
Distinct metastatic sites, *n* (%)							0.16
One (only the spine)	56 (45.2)	16 (31.4)	5 (35.7)	6 (46.2)	9 (69.2)	5 (41.7)	
Two or more	68 (54.8)	35 (68.6)	9 (64.3)	7 (53.8)	4 (30.8)	7 (58.3)	
Location of other metastases, *n* (%)							
Lung	29 (23.4)	15 (29.4)	6 (42.9)	4 (30.8)	2 (15.4)	0 (0)	0.11
Nonspinal bone	32 (25.8)	17 (33.3)	4 (28.6)	1 (7.7)	1 (7.7)	6 (50.0)	0.06
Lymph node	13 (10.5)	8 (15.7)	0 (0)	2 (15.4)	1 (7.7)	2 (16.7)	0.56
Other locations	9 (7.3)	8 (15.7)	0 (0)	1 (7.7)	0 (0)	0 (0)	0.14
Revised Tokuhashi score, mean (SD)	11.5 (1.8)	11.6 (1.3) ^#,¶,§^	13.0 (1.6) ^†,\,¶^	10.8 (1.2) ^#,¶,§^	8.8 (1.7) ^†,#,\,§^	13.7 (1.1) ^†,\,¶^	-
Tomita score, mean (SD)	4.1 (1.3)	3.7 (0.9) ^\,¶,§^	3.1 (1.2) ^\,¶^	5.6 (1.0) ^†,#,§^	5.4 (0.8) ^†,#,§^	2.5 (0.5) ^†,\,¶^	-
Preoperative systemic therapy, *n* (%)	65 (52.4)	28 (54.9)	5 (35.7)	3 (23.1)	7 (53.8)	9 (75.0)	0.07
Postoperative systemic therapy, *n* (%)	85 (68.5)	35 (68.6)	10 (71.4)	9 (69.2)	9 (69.2)	10 (83.3)	0.88

^†^*p* < 0.05 versus the Kidney group, ^#^ *p* < 0.05 versus the Thyroid group, ^\^ *p* < 0.05 versus the Low-grade sarcoma group,^¶^ *p* < 0.05 versus the Lung group, ^§^ *p* < 0.05 versus the Breast group. Abbreviations: ECOG PS, Eastern Cooperative Oncology Group performance status; SD, standard deviation.

**Table 2:** Medium to long-term clinical outcomes of spinal metastasectomy.

Groups	Total	Kidney	Thyroid	Low-Grade Sarcoma	Lung	Breast	*p*
Number of patients	124	51	14	13	13	12	-
3-year survival, %	70.2	76.5	92.9	76.9	61.5	66.7	0.45
5-year survival, %	59.9	60.6	85.7	69.2	61.5	66.7	
Median survival, months	93	70	ND	69	101	116	-
Local recurrence of the operated spinal lesion, *n* (%)	12 (9.7)	3 (5.9)	1 (7.1)	2 (15.4)	1 (7.7)	1 (8.3)	0.86
Instrumentation failure requiring revision surgery, *n* (%)	27 (21.8)	10 (19.6)	4 (28.6)	3 (23.1)	4 (30.8)	1 (8.3)	0.65
Patients who had only one metastasis in the operated spine at the time of surgery							
Number of patients	56	16	5	6	9	5	-
3-year survival	62.5	81.3	100	50.0	55.6	80.0	0.41
5-year survival	56.6	65.6	80	50.0	55.6	80.0	
3-year survival with NED, *n* (%)	24 (42.9)	9 (56.3)	4 (80.0)	1 (16.7)	4 (44.4)	3 (60.0)	0.29

Abbreviations: NED, no evidence of disease; ND, not determined.

**Table 3:** Three-year clinical outcomes of patients who survived for more than 3 years after spinal metastasectomy.

Groups	Total	Kidney	Thyroid	Low-Grade Sarcoma	Lung	Breast	*p*
Number of patients	88	39	13	10	8	8	-
Proportion of ≥3-year survivors	71.0%	76.5%	92.9%	76.9%	61.5%	66.7%	0.38
Good PS (ECOG PS: 0 or 1) 3 years after surgery, *n* (%)	80 (90.9)	35 (87.5)	11 (84.6)	10 (100)	8 (100)	7 (87.5)	0.61
Preoperative systemic therapy, *n* (%)	46 (52.3)	22 (56.4)	4 (30.8)	3 (30.0)	5 (62.5)	6 (75.0)	0.15
Postoperative systemic therapy, *n* (%)	62 (70.5)	28 (71.8)	9 (69.2)	6 (60.0)	6 (75.0)	7 (87.5)	0.76
Antiresorptive therapy, *n* (%)	30 (34.1)	16 (40.0)	4 (30.8)	1 (10.0)	1 (12.5)	7 (87.5)	<0.05
Disease progression of BMs except for the operated spine lesions, *n* (%)	27 (30.7)	14 (35.0)	4 (30.8)	3 (30.0)	1 (12.5)	3 (37.5)	0.79
Additional excisional surgery for other BMs, *n* (% of patients with disease progression of BMs)	12 (44.4)	7 (50.0)	3 (75.0)	0 (0)	0 (0)	0 (0)	0.12
Disease progression of non-skeletal metastases, *n* (%)	36 (40.9)	19 (47.5)	2 (15.4)	8 (80.0)	2 (25.0)	2 (25.0)	<0.05
Additional excisional surgery for non-skeletal lesions, *n* (% of patients with disease progression of non-skeletal metastases)	8 (25.0)	7 (36.8)	0 (0)	3 (37.5)	0 (0)	0 (0)	0.53

Abbreviations: BM, bone metastasis; ECOG PS, Eastern Cooperative Oncology Group performance status.

## Data Availability

The data that support the findings of this study are available on request from the corresponding author. The data are not publicly available due to privacy or ethical restrictions.
